# Changes in patient care through flexible and integrated treatment programs in German psychiatric hospitals: meta-analyses based on a series of controlled claims-based cohort studies

**DOI:** 10.1186/s12888-024-05500-0

**Published:** 2024-01-26

**Authors:** Anne Neumann, Jochen Schmitt, Martin Seifert, Roman Kliemt, Stefanie March, Dennis Häckl, Enno Swart, Andrea Pfennig, Fabian Baum

**Affiliations:** 1 Center of Evidence-Based Health Care (ZEGV), Medizinische Fakultät Carl Gustav Carus, Technische Universität Dresden, Fetscherstraße 74, 01307 Dresden, Germany; 2 WIG2 Scientific Institute for Health Economics and Health System Research Leipzig, Leipzig, Germany; 3 Institute of Social Medicine and Health Services Research, Medical Faculty, Otto-Von-Guericke-University, Magdeburg, Germany; 4 Department of Social Work, Health and Media, Hochschule Magdeburg-Stendal, Magdeburg, Germany; 5 Department of Psychiatry and Psychotherapy, Carl Gustav Carus University Hospital, Technische Universität Dresden, Dresden, Germany

**Keywords:** Claims data, Mental health care, Global treatment budget, Inpatient and outpatient treatment, Treatment continuity, FIT hospital

## Abstract

**Background:**

Global treatment budgets, i.e. predefined budgets for patients treated in hospital independent of the setting within the hospital, together with flexible and integrated treatment (FIT) have been introduced in some German psychiatric hospitals since 2013. We investigated pooled changes in inpatient, day-care, outpatient treatment, and continuity of care for patients with mental disorders in 12 FIT-hospitals.

**Methods:**

We conducted a series of 12 controlled cohort studies regarding FIT hospitals using anonymized patient claims data from more than 70 German statutory health insurance funds. Each study compared one FIT-hospital to matched patients from equivalent non-FIT-hospitals (routine care). We included only those patients without treatment in the respective hospital within two years prior to first hospital treatment (either FIT or routine care). We contrasted results between the year prior to with the first and second year after patient’s first treatment (treatment continuity: only group comparison) using multivariate multi-level models. To approximate the difference-in-difference effect in the meta-analysis, we used the interaction terms *group* (FIT hospital vs. routine care) x *time* (year before vs. first or second patient year after study inclusion) in the Poisson models.

**Results:**

The 12 studies included 36,069 patients with 2,358 patients from a Department of child and adolescent psychiatry. The pooled effect revealed a 5.1 days lower increase in inpatient treatment in FIT-hospitals during the first patient year compared to routine care. Results were statistically significant for adult care FIT-hospitals but not for child and adolescent FIT-hospitals. Utilization of day-care treatment increased more in most FIT-hospitals during the first year, while outpatient contacts increased in some and decreased in others. The odds of treatment continuity increased by 1.4 in FIT-hospitals compared to non-FIT-hospitals.

**Conclusions:**

Global treatment budgets lead to the intended changes in mental health care in the majority of FIT-hospitals compared to routine care in this large real-world evidence study from Germany. For child and adolescent psychiatry, more evidence is needed to draw firm conclusions.

**Trial registration:**

This study was registered in the database “Health Services Research Germany” (trial number: VVfD_EVA64_15_003713).

**Supplementary Information:**

The online version contains supplementary material available at 10.1186/s12888-024-05500-0.

## Background

Mental disorders are diverse and patients require needs-tailored treatment options. Adequate and patient-centered treatment is therefore inevitable [1]. Hence, hospitals treating mentally ill patients should be enabled to offer the best possible treatment tailored to the patient’s need. In Germany, there is a strong separation between inpatient and outpatient treatment (sectors) due to different financing and responsibilities, which is historically grown [2, 3]. However, patients with mental disorders need continuous and patient-centered treatment. Treatment in German psychiatric hospitals is mostly conducted as inpatient, day-care or intensive and complex outpatient treatment (PIA, Psychiatrische Institutsambulanz) (setting). In addition, innovative treatment forms, such as home treatment, have been introduced in German psychiatric hospitals. Substantial boundaries between those settings are incentivizing hospitals to prolong inpatient treatment as outpatient or day-care treatment is sometimes insufficiently and inpatient care relatively well remunerated [4–7]. This strict separation between sectors, i.e. inpatient vs. outpatient treatment, and its fragmented financing obstruct cross-sectoral treatment continuity and advances in mental health care provision [2, 8]. Patients in Germany are, therefore, at risk to fall out of treatment after inpatient care [2, 9]. In sum, the complex treatment and partly misincentivized pathways across settings and sectors, e.g. incentives to longer inpatient treatment as optimal and lack of flexible and integrated treatment structures such as assertive community treatment, hinders adequate treatment within the hospital and beyond hospital treatment, e.g. reduced adherence and therapeutic outcomes of overstrained patients due to complexity of health care provision.

The strict budget and care barrier between outpatient and inpatient care is of great importance as changes in care models in Germany are based on policy decisions driven by hospitals or mental health professionals working with policy makers to improve patient-centered care [3, 10]. Being aware of this situation, a few psychiatric hospitals introduced different treatment and financing options. Already in 2003, regional psychiatry budgets were introduced in the northern Federal State Schleswig Holstein allowing cross-setting financing and treatment [11]. In addition, several contracts for integrated care (“integrierte Versorgung”—IV) have been established on a regional and/or disease-specific basis [12, 13]. Based on the positive experiences from those care concepts [14–17] and the urge to provide a federal solution for the entire federal republic, a new law was introduced. Since 2012, the German law (§ 64b Social Code book (SGB) V) has enabled hospitals and statutory health insurance (SHI) funds to establish time-limited contracts allowing global treatment budgets and flexible and integrated treatment (FIT) programs [18]. FIT programs aim at offering patient-centered and needs-adapted treatment options. In contrast to most other FIT programs internationally, the FIT programs mentioned here are offered by hospital-based teams to patients treated in psychiatric hospitals using a Global Treatment Budget (GTB) [19].

The GTB is a combination of block contracts and per-capita financing where the hospital receives a total budget for all forms of inpatient and hospital-based outpatient care [14, 20, 21]. Therefore, FIT hospitals are provided with a fixed monetary amount for the treatment of their patients, independent from the hospital setting in which treatment actually takes place. The main aims of all FIT hospitals are to strengthen non-inpatient treatment options, such as home treatment and outpatient treatment in the hospital, to optimize cross-setting and cross-sectoral treatment, and to expand multi-professional collaboration [22]. Global budgets leave it to the decision of the hospital and service providers to decide which treatment form (inpatient, day care, outpatient, home treatment, etc.) a patient is treated best. The payment per capita is not based on the setting of treatment, as the global treatment budget is fix independent from where and how the patient is treated. It, therefore, depends on the experiences and focus of the FIT hospitals, which specific aspects of integrated care is in the center of treatment. In usual care, on the other hand, the budget refers exclusively to the inpatient sector, while outpatient revenues in the hospital are excluded from the budget. Hence, the system in usual care currently provides no incentive to treat across settings or to shift to non-inpatient treatments.

On the other hand, the change from a daily- and performance-based financing to a GTB is identified as a key driver towards a more flexible and integrated treatment [23]. Such GTBs, therefore, allow for strengthening innovative integrated treatment options provided by multi-professional teams, such as Assertive Community Treatment, Home-Treatment [24], Crisis Resolution Teams [25], or a stronger focus on day-care treatment allowing more need-adapted, cross-sectoral service delivery [26]. Most experts regard integrated and flexible treatment programs as a fundamental basis for adequate contemporary mental health care [1, 19, 27]. The specific treatment plans were adapted to the profile and situation of the specific hospital, region and patients and were therefore different between FIT hospitals. Nonetheless, the implementation of a GTB and alternative treatment options are common in all FIT hospitals investigated here. Evidence on GTB and previous aligned concepts showed advantages of the new care concepts compared to usual care, e.g. regarding shorter duration of inpatient days [17, 28–30], patient-centered outcomes [31], conditions for successful GTB implementation [32], and identified core mechanisms of change in FIT hospitals [23].

So far, 22 FIT hospitals have been established in Germany. They differ regarding size, focus, content, treatment structures and processes, depending on local conditions and experiences [19, 33]. All FIT hospitals, however, have in common that they seek to offer continuous, flexible and integrated forms of treatment rather than short periods of care with the focus on inpatient treatment [19]. As FIT hospitals were introduced on a time-limited basis, mostly for a duration of eight years with an option of prolongation, and should provide evidence to aid the decision on whether to implement such an alternative financing system into routine care, all FIT hospitals need to be evaluated by independent researchers by law [34]. Eighteen of the above-mentioned 22 FIT hospitals were included in a nationwide evaluation (EVA64). By November 2021, 12 of the 18 FIT hospitals have already received final evaluation reports [35]. FIT hospitals differed concerning their situation before the onset of FIT programs as some had already tested alternative financing and treatment options similar to those of FIT programs with FIT-like pre-existing contracts [33]. Others, however, freshly started FIT programs from routine care. Differences in the starting condition influenced the transition into FIT care concerning inpatient, day-care and outpatient treatment within the hospital [28, 29]. A transition period from routine care to FIT structures of about two years is expected. Therefore, it is essential to investigate results covering more than two years after FIT start. Second, treatment efforts are often highest during patient’s first hospital treatment (e.g. first acute crisis) compared to subsequent treatments. However, a follow-up for more than the first year provided evidence for the long-term results of such programs. In addition, treatment continuation is also an important factor for successful long-term outcomes. So far, no study has included a recruitment phase of two years or more, considered more than two patient years (follow-up time) and summarized results over a large number of FIT hospitals.

The aim of our analysis, focusing on the above-mentioned clinical aims of the GTB, was to investigate the number of inpatient and day-care days as well as outpatient contacts at the hospital (PIA) and the treatment continuity of patients treated in FIT hospitals compared to usual care.

## Methods

### Study design and inclusion and exclusion criteria

We conducted a series of controlled cohort studies regarding FIT hospitals using anonymized patient claims data [36] from more than 70 German statutory health insurance (SHI) funds, which covered more than 70 percent of all patients with mental disorders of FIT and control hospitals. Each of the controlled cohort studies evaluates one FIT hospital in the study EVA64 [37]. We described the design of the EVA64 study elsewhere [37]. To compare results across several FIT hospitals, we included those patients who had at least two calendar years of follow-up (plus those who died in the year of interest), and who were treated in the FIT or control hospital within the first three years after FIT program start (either inpatient, day-care or PIA). In addition, the included FIT hospital needed to have experienced eight years of FIT treatment by the end of 2021. We excluded patients who received treatment in the hospital within the two years prior to study inclusion to have comparable starting conditions between the patients and to investigate the effect of FIT hospitals in patients with initial treatment needs. Patients from 12 FIT hospitals fulfilled the inclusion criteria with two hospitals also including children (Department of child and adolescent psychiatry, CAP).

**Fig. 1:**
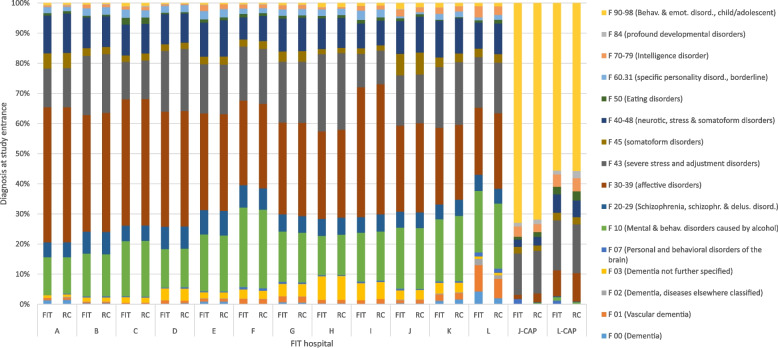
Proportion of diagnoses (references case) in FIT hospitals and respective controls More than one diagnosis was considered if index case was during PIA (psychiatric outpatient department) treatment (no main diagnosis identifiable within data), while only the main diagnosis was considered if index case was during inpatient or day-care stay; FIT = flexible and integrated treatment = those hospitals with innovative financing and treatment forms (intervention group); RC = routine care; CAP = Department of child and adolescent psychiatry.* = FIT hospital with FIT-like pre-existing contract

### Matching of control group (routine care)

We compared the results of patients from each FIT hospital with patients from routine care following an intention-to-treat approach, i.e. patients remained in the treatment (FIT) or control group (routine care) according to their first contact after the start of the FIT program. We homogenized both samples of patients via a two-fold matching procedure on hospital and patient level in order to have comparable hospitals with comparable patients for each FIT hospital. On the hospital level, we allocated up to 10 hospitals (control hospitals) to each FIT hospital. We based hospital allocation on an a priori defined ranking using knockout criteria (i.e. same region, institutionalized structure such as specialist departments, and PIA), patients criteria (i.e., number of cases per diagnosis) with a weighting of 50%, structural hospital features (e.g. number of beds or personnel) with a weighting of 25%, and regional factors (e.g. unemployment rate) with a weighting of 25%. For more details on the selection of control hospitals, see the control hospital’s methods description [38] and the EVA64 study protocol [37]. On the patient level, we matched patients (1:1) exactly according to the variables: year of study inclusion and psychiatric diagnosis at study inclusion. In addition, we applied propensity score matching (nearest neighbor, caliper = 0.25) on the variables: age at study inclusion, sex, and health care utilization before study inclusion (in the area of mental health care: amount of inpatient care, day-care, and number of PIA and resident physicians contacts). If less than 95% of patients in the FIT hospitals could be matched, we applied another matching round ignoring the mental health diagnosis at study inclusion during the exact matching. Further, we increased the caliper in the propensity score matching step by step (by 0.01) until at least 95% of the patients from the FIT hospitals found a matching partner.

### Outcomes and data

We analyzed the *duration of inpatient care*, the *duration of day-care treatment*, *outpatient treatment within hospitals* and *cross-sectoral treatment continuity* to measure the anticipated change in patient treatment within the hospital after initiation of a global budget and the FIT program in FIT hospitals. *Duration of inpatient* and *day-care treatment* were defined as the cumulated number of days treated either as inpatient or as day-care. *Outpatient treatment within hospitals* was defined as the number of contacts in PIA care. As data on PIA were not available before 2013, we excluded those patients who were included in the analysis before 2014 from the year prior to study inclusion for this analysis. *Cross-sectoral treatment continuity* was defined as the percentage of inpatients receiving outpatient treatment continuation, i.e. the percentage of patients who were treated in the hospital due to a psychiatric diagnosis and had at least one contact in PIA, with a resident medical specialist for adult or child and adolescent psychiatry, or with a psychotherapist within 30 days after hospital discharge. Contrary to the other analyses, the statistical units of *cross-sectoral treatment continuity* were not the number of patients but the number of inpatient cases within the respective year with at least 30 days of follow-up. Following a pre-post design, we compared patient results between the patient individual year before and the first and second year after study inclusion. As we expected that the anticipated reduced inpatient care would be compensated through either increase of day-care or PIA treatment, we analyzed the relationship between day-care and PIA treatment. Due to the retrospective nature of the study and the analysis of anonymous data, the ethics committee of the Otto-von-Guericke-University at the Medical Faculty and University Hospital Magdeburg confirmed that no ethical approval was necessary. Data were handled, analyzed and reported according to Good Epidemiological Practice (GEP) [39], Good Practice of Secondary Data Analysis (GPS) [40], the German Reporting Standard for Secondary Data Analyses, Version 2 (STROSA 2) [41].

### Statistical analysis

We applied generalized linear Poisson models, adjusted by age, sex, diagnosis at study entry, comorbidities, setting at study entry (inpatients vs. PIA) and severe mental disorders, to estimate outcome parameters. We measured comorbidities by summing up the number of Elixhauser groups [42]. We defined severe mental disorders as being diagnosed with any of the following diagnoses (ICD-10): F20.X-F22.X (schizophrenia/schizophrenic disorders), F25.X (schizoaffective disorders), F30.X (mania), F31.X (bipolar affective disorder), F32.2-F32.3 (major depressive episodes), F33.X (recurrent depressive disorder), F41.X (other anxiety disorders), F42.X (obsessive–compulsive disorder), or F60.31 (specific personality disorder of the borderline type) (based on [43]).

To approximate the difference-in-difference (DiD) effect in the meta-analysis, we used the interaction terms *group* (FIT hospital vs. routine care) x *time* (year before vs. first or second patient year after study inclusion) in the Poisson models. The DiD estimate compares the average change in the outcome over time for the FIT hospital in comparison to the average change in time in routine care. Thus, greater increase over time in the FIT hospital compared to routine care are associated with a positive DiD estimate and vice versa [28]. We used the DiD coefficient for every single FIT hospital in a meta-analysis utilizing the R package metaphor [44]. For treatment continuity, we compared the odds for treatment continuity between FIT hospitals and routine care calculating odds ratios (OR). The meta-analyses followed a multi-variate multi-level approach [44, 45].

The forest plots showing the results of the meta-analyses displayed the comparison between the year before and the first or second year after patient individual study inclusion. To investigate the influence of CAP in the overall meta-analysis, we conducted sub-group meta-analyses for adult psychiatry only. In addition, we carried out a meta-regression to investigate the impact of precursor contracts in FIT hospitals. We included a predictor variable into the meta-analysis which dummy coded hospitals with precursor contracts (0 = no existing contract vs. 1 = existing contract). The result of the meta-regression gives an estimate on how the pooled estimates in FIT hospitals with precursor contracts differs when contrasted against those without such a contract. To analyze the association between day-care and PIA treatment, we used a Pearson correlation coefficient on the level of hospitals. We applied 5% as the level of significance and conducted all statistical analyses using the statistical software R V.3.3.2 [46].

## Results

### Study population

During the first patient year, a total of 36,069 patients from 12 FIT hospitals and their matched group from routine care (RC) were included in these analyses, with 18,062 being in the FIT hospital and 18,007 in the RC group (Table 1).

**Table 1:** Study population

FIT hospital	Number of patients				Mean age at study inclusion (years)		Percentage women (%)		Start of FIT program (year)		FIT-like pre-existing contract	
	FIT		RC		FIT	RC	FIT	RC	FIT	RC	FIT	RC
	1st yr	2nd yr	1st yr	2nd yr								
**A**	928	906	925	905	48.2	48.6	62.8	61.9	2014	n.a	No	n.a
**B**^a^	2,152	2,094	2,146	2,092	47.6	47.3	54.8	55.0	2013		Yes	
**C**^a^	1,624	1,578	1,616	1,574	46.9	46.7	56.2	57.6	2013		Yes	
**D**^a^	1,262	1,201	1,257	1,205	49.8	48.9	56.3	59.2	2013		Yes	
**E**^a^	1,826	1,757	1,815	1,763	49.4	48.2	57.0	55.2	2013		Yes	
**F**^a^	1,041	1,003	1,039	1,003	48.9	48.4	51.1	50.0	2014		Yes	
**G**^a^	1,541	1,479	1,541	1,477	48.8	49.1	58.1	57.4	2013		Yes	
**H**	2,374	2,231	2,377	2,248	48.3	48.4	54.5	55.7	2014		No	
**I**	1,021	973	1,012	967	49.5	49.5	56.3	55.4	2014		No	
**J**	980	938	974	942	50.3	48.9	62.8	70.6	2014		No	
**K**	1,359	1,281	1,359	1,290	53.8	53.1	54.1	53.6	2014		No	
**L**^a^	772	730	770	730	51.2	52.5	51.0	51.8	2014		Yes	
**J—CAP**	734	734	717	716	11.0	11.2	42.4	39.2	2014		No	
**L**^a^**—CAP**	448	447	459	459	12.1	12.4	43.5	45.1	2014		Yes	
**Total**	18,062	17,352	18,007	17,371								

*FIT* Flexible and integrated treatment = those hospitals with innovative financing and treatment forms (intervention group), *RC* Routine care, *1st yr.* First patient year, *2nd yr.* Second patient year, *CAP* Department of child and adolescent psychiatry, *n.a.* Not applicable

^a^FIT hospital with FIT-like pre-existing contract

As we could not follow some patients for two years, there is a difference of the sample size between FIT hospitals and the RC group in the first patient year (in comparison to the patient matching where we only needed data for one year). The difference between first and second patient year is due to the different number of people dying in both groups in the first patient year. Of the 36,069 patients, 2,358 patients (FIT: 1,182; RC: 1,176) were treated in a CAP. During the second patient year, 34,723 patients could be included in the analysis. Thus, 1,346 patients from the first patient year could not provide data for the second patient year due to death or change of SHI fund. Age and female-to-male ratios were comparable between FIT hospitals and respective RC groups. Each FIT hospital started its FIT program in either 2013 or 2014. Seven of the 12 FIT hospitals already employed pre-existing FIT-like conditions before the onset of the FIT program using either a regional budget for mental health, a contract for integrated care or both [33]. Five FIT hospitals freshly started from routine care. The psychiatric diagnoses which led to the index treatment in either FIT or RC hospitals were comparable between both groups with most patients being diagnosed with affective disorders (ICD-10: F30-39) among adults and with behavioral and emotional disorders which started in childhood or adolescence (F90-98) among children and adolescents (Fig. 1).

### Inpatient days

**Fig. 2:**
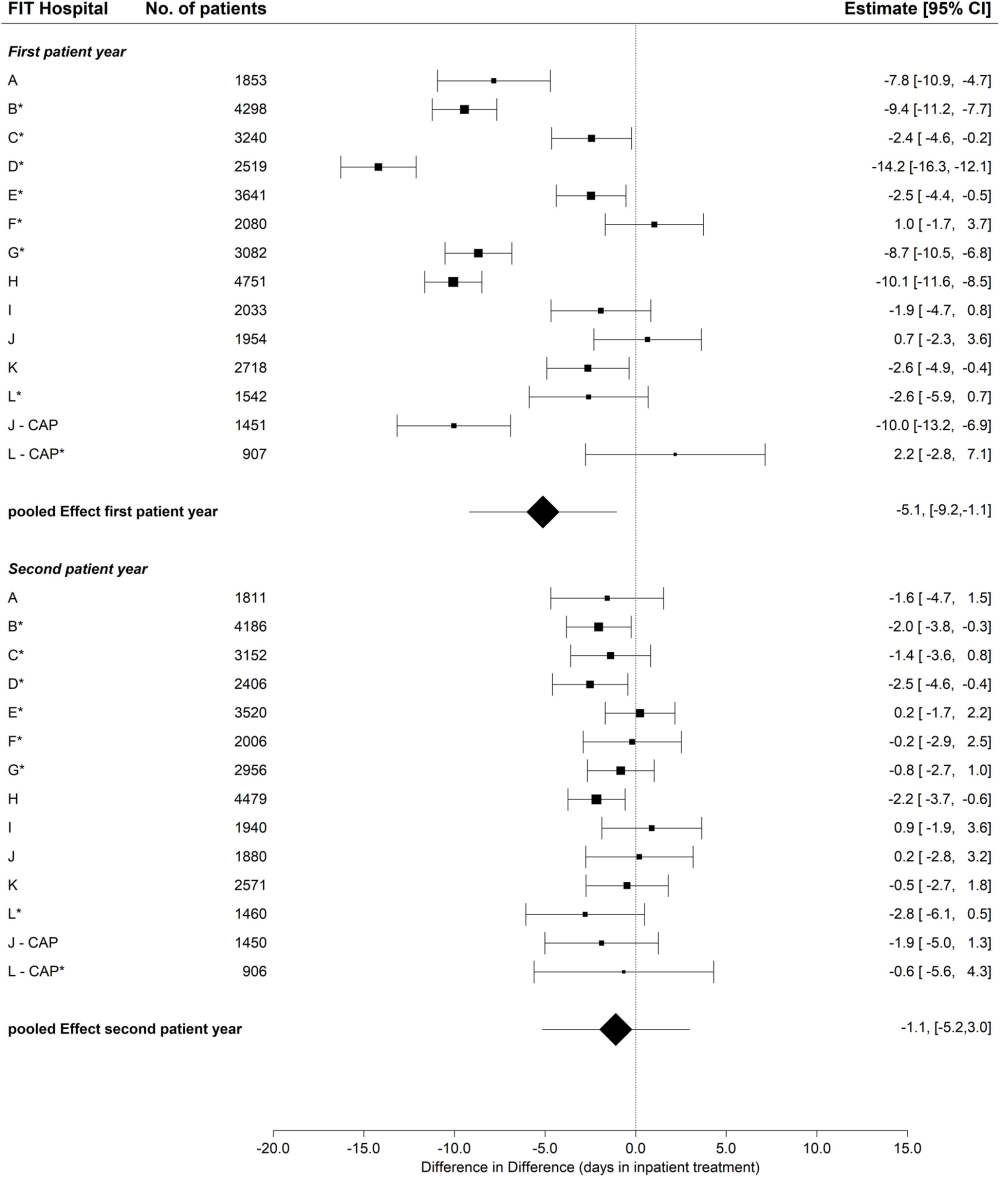
Change in inpatient days, first and second patient year, FIT vs. routine hospitals CAP = Department of child and adolescent psychiatry. * = FIT hospital with FIT-like pre-existing contract. Difference in Difference = The difference in difference (DiD) estimate compares the average change in the outcome over time for the FIT hospital in comparison to the average change in time in routine care. Thus, greater increase over time in the FIT hospital compared to routine care are associated with a positive DiD estimate and vice versa

The majority of FIT hospitals (n_PSY_ = 10; n_CAP_ = 1) showed a smaller increase of inpatient treatment days during the first patient year comparing the years before and after study inclusion and FIT hospitals with routine care (group x time) (Fig. 2).

Eight estimates were statistically significant. The pooled estimator revealed a 5.1 days (95% CI -9.2, -1.1) lower increase in inpatient treatment compared to routine care. In the second patient year, the increase in inpatient treatment days continued to be lower in the FIT hospitals than in routine care (-1.1 inpatient days; 95% CI: -5.2, 3.0). However, this difference was smaller than in the first year and only statistically significant in three FIT hospitals. The sub-group analysis without CAP showed similar results, whereas the sub-group analysis with CAP only revealed very wide confidence intervals due to the heterogeneous effects of the two CAPs (Table 2 and Fig. S1). The descriptive data can be found in Table S1.

**Table 2:** Pooled effects, inpatient care, PIA contacts and treatment continuity with, without CAP and CAP only

	**Pooled estimate**					
**Outcome**	**with CAP (95% CI)**		**without CAP(95% CI)**		**CAP only (95% CI)**	
	1st yr	2nd yr	1st yr	2nd yr	1st yr	2nd yr
Inpatient care (days)	-5.1 (-9.2, -1.1)	-1.1 (-5.2, 3.0)	-5.2 (-9.2,-1.2)	-1.1 (-5.1, 2.9)	-4.2 (-25.6, 17.1)	-1.9 (-5.0, 1.3)
PIA contacts	0.3 (-1.7, 2.3)	0.1 (-1.9, 2.1)	0.6 (-1.4, 2.6)	0.1 (-1.9, 2.2)	-1.2 (-7.9, 5.5)	-0.3 (-7.0, 6.4)
Treatment continuity (odds ratio)	1.4 (1.1, 1.8)	1.2 (0.9, 1.6)	1.3 (1.0, 1.8)	1.2 (0.9, 1.6)	-0.2 (-1.5, 1.2)	0.7 (-0.7, 2.0)

Due to the low number of cases among CAP cases for the outcome day care, the pooled estimate generally included no patients from the CAP for this outcome and did not need to be considered here

*CAP* Department of child and adolescent psychiatry, *1st yr.* First patient year, *2nd yr.* second patient year, *PIA* Psychiatric outpatient department at hospital

### Day-care days

**Fig. 3:**
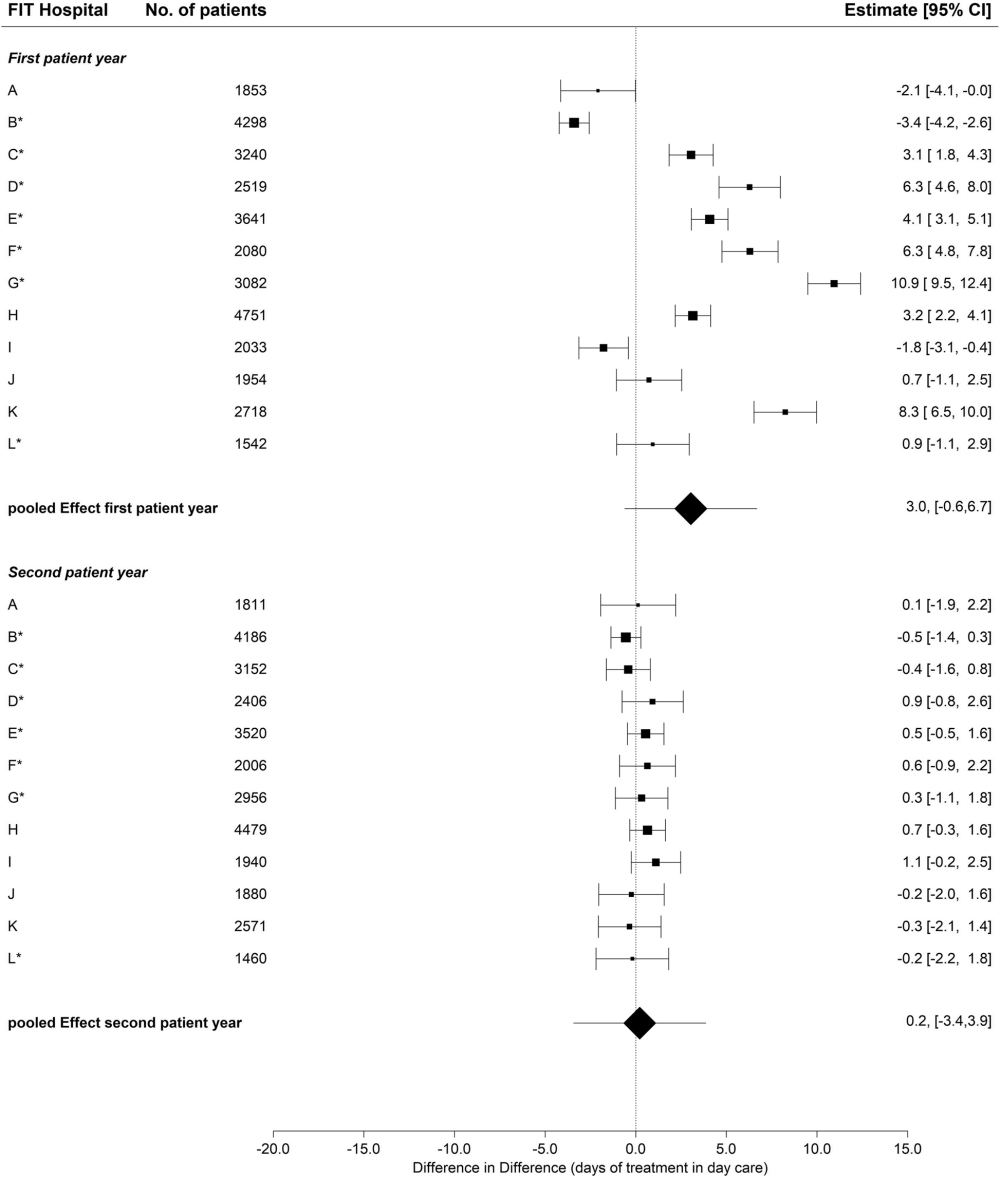
Change in day-care days, first and second patient year, FIT vs. routine hospitals * = FIT hospital with FIT-like pre-existing contract. Due to the low number of cases, the CAP (Department of child and adolescent psychiatry) was excluded from this analysis. Difference in Difference = The difference in difference (DiD) estimate compares the average change in the outcome over time for the FIT hospital in comparison to the average change in time in routine care. Thus, greater increase over time in the FIT hospital compared to routine care are associated with a positive DiD estimate and vice versa

Compared to inpatient treatment days, the majority of FIT hospitals showed a larger increase in the number of day-care treatment days during the first patient year compared to routine care (Fig. 3). Across all FIT hospitals, FIT hospitals had a greater increase (95% CI: -0.6, 6.7) in the number of day-care treatment days by 3.0 days; however, this difference was not statistically significant. In the second patient year, there were hardly any differences between FIT hospitals and routine care. The numerical differences between the two groups were very small for all FIT hospitals. The descriptive data can be found in Table S2.

### PIA contacts

**Fig. 4:**
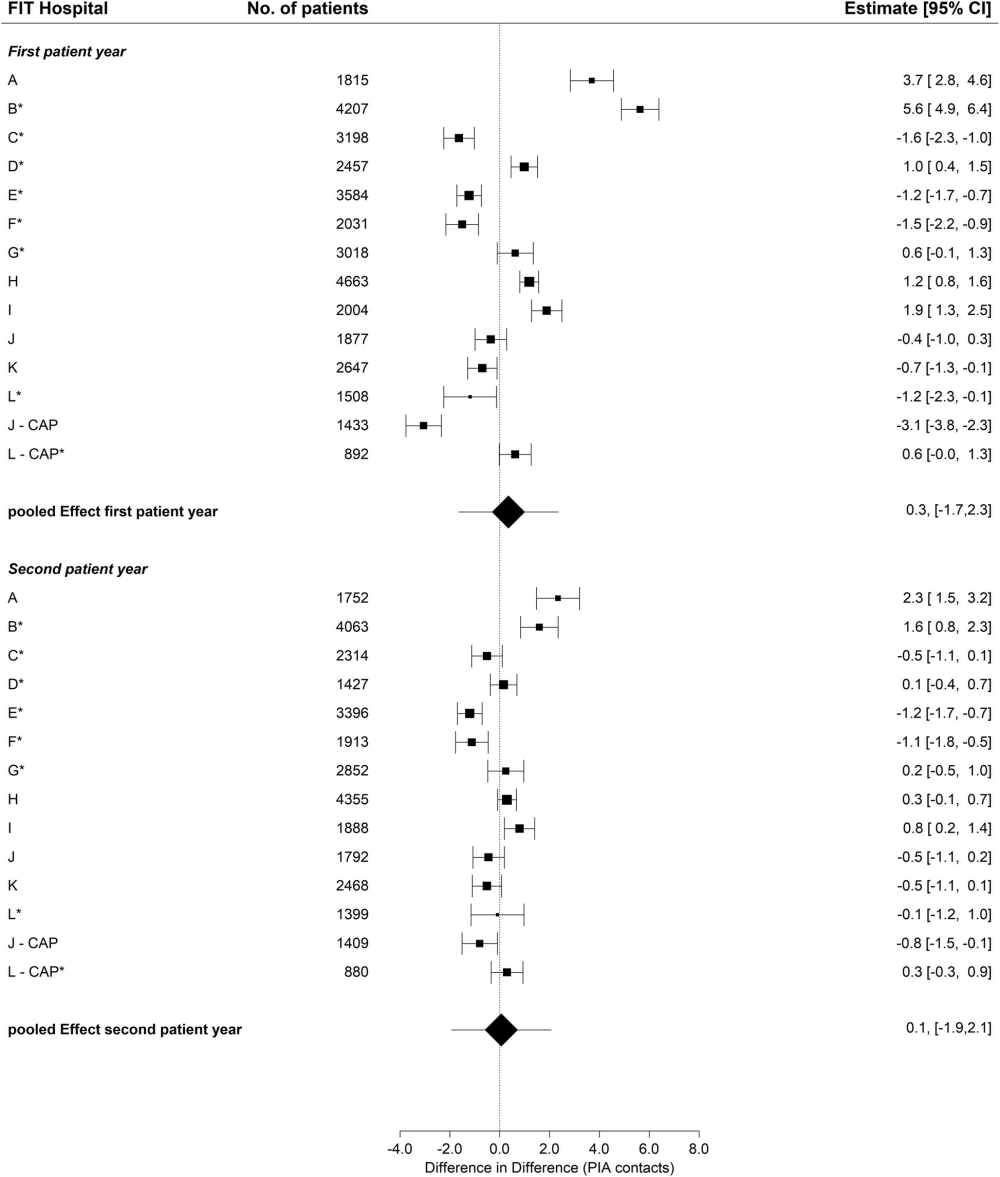
Change in number of PIA contacts, first and second patient year, FIT vs. routine hospitals CAP = Department of child and adolescent psychiatry. * = FIT hospital with FIT-like pre-existing contract. Difference in Difference = The difference in difference (DiD) estimate compares the average change in the outcome over time for the FIT hospital in comparison to the average change in time in routine care. Thus, greater increase over time in the FIT hospital compared to routine care are associated with a positive DiD estimate and vice versa

In the first patient year, half of the FIT hospitals showed a larger and the other half a smaller increase in PIA contacts compared to routine care (Fig. 4). In the second patient year, the increase in PIA contacts, analogous to the analysis of day-care treatment days, differed hardly between the FIT hospitals compared to routine care. However, in the three FIT hospitals that did not show an increase in day-care days but a strong increase in PIA contacts (A, B and I), the number of PIA contacts in the second year was also (statistically significantly) greater than in routine care. The sub-group analysis without CAP revealed slightly more PIA contacts among patients in the FIT hospitals in the first patient year compared to the analysis with CAP (Table 2 and Fig. S2). However, this difference was still not statistically significant (pooled estimate 1st year: 0.6, 95% CI: -1.4, 2.6). The descriptive data can be found in Table S3.

### Association between day-care days and PIA contacts

In FIT hospitals that had a statistically significant increase in PIA contacts in both years (Fig. 4), the utilization of day-care treatment was statistically significantly lower in the first year compared to standard care (Fig. 3). This observation also held true in the reverse case, so that those FIT hospitals with statistically significantly fewer PIA contacts in both years claimed statistically significantly more day-care treatment days in the first patient year. The correlation estimate assessing the association between day-care and PIA revealed an inverse association during the first patient year (*r* = -0.46, *p*-value: 0.14) meaning the higher the number of days in day-care treatment the lower the number of PIA contacts and vice versa (Table 3). This association, however, was not statistically significant. During the second patient year, the association was weak (*r* = 0.15, *p*-value: 0.63).

**Table 3:** Correlation, day-care days and PIA contacts

	*r*	*p-value*	df
First patient year	-0.46	0.135	10
Second patient year	0.15	0.631	10

*r* Correlation, *df* Degree of freedom

### Treatment continuity

**Fig. 5:**
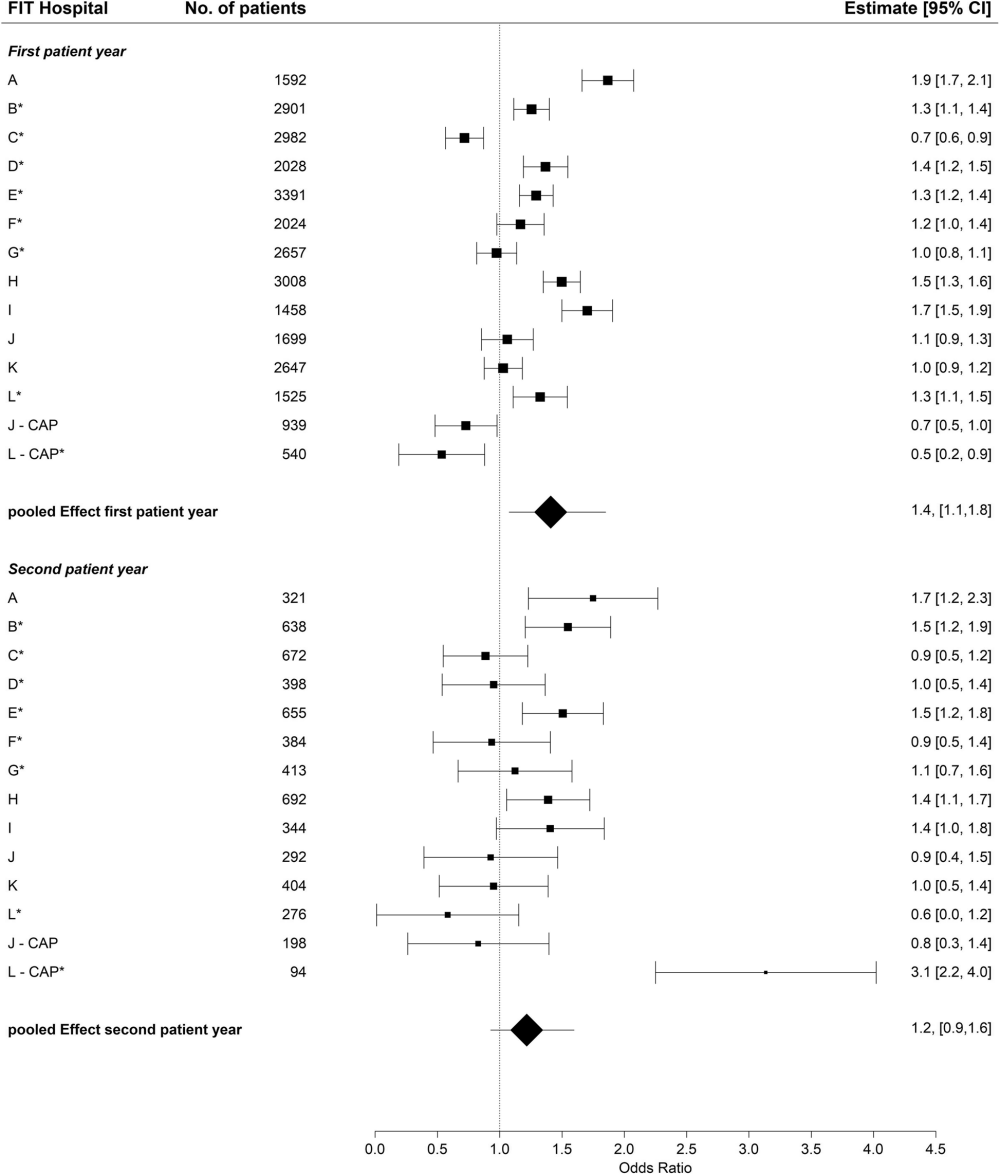
Change in treatment continuity, first and second patient year, FIT vs. routine hospitals, Odds Ratios Treatment continuity = outpatient treatment within 30 days after hospital discharge. CAP = Department of child and adolescent psychiatry, number of cases = number of cases with inpatient treatment and at least 30 days of follow-up time, * = FIT hospital with FIT-like pre-existing contract, Difference in Difference = The difference in difference (DiD) estimate compares the average change in the outcome over time for the FIT hospital in comparison to the average change in time in routine care. Thus, greater increase over time in the FIT hospital compared to routine care are associated with a positive DiD estimate and vice versa

The pooled estimator of treatment continuity was greater in the FIT hospitals than in routine care, in both the first (OR: 1.4, 95% CI: 1.1, 1.8) and second patient year (OR: 1.2, 95% CI: 0.9, 1.6) (Fig. 5). In the majority of the FIT hospitals, treatment continuity was greater than in routine care. In addition to the hospitals that already provided more PIA contacts (see Fig. 4), which is also counted as outpatient contact, the FIT hospitals E and L also showed an additional increased continuity of treatment compared with routine care despite fewer PIA contacts. Patients treated at FIT hospital E had more “at-least-one contacts” within 30 days after inpatient treatment in a PIA or with a psychotherapist compared to routine care during the first patient year. On the other hand, patients treated at FIT hospital L had more “at-least-one contacts” with a resident medical specialist for adult or child and adolescent psychiatry (Table 4). The sub-group analysis without CAP showed a slightly lower treatment continuity for the first patient year (Table 2 and Fig. S3). This result was, in contrary to the overall meta-analysis, no longer statistically significant (OR: 1.3, 95% CI: 1.0, 1.8). The descriptive data can be found in Table S4.

**Table 4:** Setting of treatment continuity

Differences of % between FIT and RC Patients with at least one contact to … within 30 days after hospital discharge	PIA		Resident medical specialist for adult or child and adolescent psychiatry		Psychotherapist	
	FIT vs. RC		FIT vs. RC		FIT vs. RC	
**FIT hospital**	1st yr	2nd yr	1st yr	2nd yr	1st yr	2nd yr
**A**	+ 16.9	+ 19.9	+ 5.0	-3,9	+ 2.6	+ 0.3
**B**^a^	+ 18.3	+ 16.9	-9.6	-4.4	-0.9	+ 1.7
**C**^a^	-5.4	-2.5	-4.2	-2.6	+ 1.5	+ 3.9
**D**^a^	+ 17.0	+ 11.0	-7.5	-8.7	-1.8	-1.9
**E**^a^	+ 4.8	-0.9	-0.6	+ 5.2	+ 3.2	+ 2.4
**F**^a^	+ 10.7	+ 3.0	-5.5	+ 0.7	+ 1.3	+ 4.5
**G**^a^	+ 7.7	+ 6.9	-7.3	-4.9	-0.5	-3.8
**H**	+ 18.7	+ 18.7	-2.5	-10.5	-0.1	-8.0
**I**	+ 10.8	+ 15.3	+ 2.9	-0.2	-1.8	-2.5
**J**	+ 0.8	+ 7.7	+ 1.4	-6.7	-2.1	-3.8
**K**	+ 2.9	-6.4	+ 1.5	+ 6.8	-1.4	-5.1
**L**^a^	-2.4	-8.7	+ 12.6	-0.1	-4.3	-5.1
**J—CAP**	-5.5	-2.2	+ 1.3	+ 3.9	-6.4	+ 0.2
**L**^a^**—CAP**	-0.7	-4.8	-4.4	+ 17.4	-11.1	+ 17.4

*FIT* Flexible and integrated treatment = those hospitals with innovative financing and treatment forms (intervention group), *RC* Routine care, *PIA* Psychiatric outpatient department at hospital, *1st yr.* First patient year, *2nd yr.* Second patient year, *CAP* Department of child and adolescent psychiatry

^a^FIT hospital with FIT-like pre-existing contract

### Influence of FIT-like pre-existing contracts

We also investigated the influence of FIT-like pre-existing contracts on all four above described outcomes (Table 5). Our results indicate that there is no significant difference in the pooled estimate in all four outcomes between those FIT hospitals with such precursor contract in comparison to those without.

**Table 5:** Meta-regression effect sizes of FIT-like pre-existing contracts

Outcome	Slope estimate	*p*-value
Inpatient care days	-0.8	0.771
Day-care days	-2.4	0.370
PIA contacts	-0.2	0.885
Treatment continuity	0.1	0.562

## Discussion

### Principle findings

Our results indicate that implementing a global financing structure together with innovative integrated treatment programs leads to reduced inpatient days, increased non-inpatient treatment and enhanced treatment continuity among adult patients treated in psychiatric hospitals in Germany.

Our meta-analysis aimed at comparing different FIT hospitals in Germany using the same methodology across all FIT hospitals to gain an overall estimate of FIT hospitals’ effectiveness. However, each FIT hospital is unique with individual circumstances (e.g. treated patients, local and regional partners and infrastructure as well as FIT experience). Thus, there is no all-encompassing FIT model that may be generalized over sites making cross-hospital comparison difficult [26]. The aim of our research was to provide on the one hand an overview of each FIT hospital in comparison to other FIT hospitals and on the other hand, calculate a pooled estimate for a general message keeping in mind that FIT hospital circumstances vary.

#### Inpatient, day-care and PIA treatment

Inpatient treatment days were avoided in the majority of FIT hospitals, especially in the first patient year. In agreement with our results, studies showed that the length of inpatient stays was reduced in flexible integrated care programs (which share components of FIT care) compared to routine care [47]. Lambert et al. [48] found a reduction of psychiatric inpatient days in a group of patients with schizophrenia treated by an assertive community treatment (ACT)-based integrated care program compared to routine care. Another study analyzing the average length of hospital stay among older patients in FIT hospitals also showed shorter hospital length of stay, especially among those FIT hospitals that exclusively treat FIT patients [49].

The flexibility gained by the hospital leads to implementation of treatment concepts developed by the hospital. These treatment concepts differ in part in design and implementation between the FIT hospitals, as can be seen, among other things, from the different effects on the day-care treatment days and PIA contacts. Day-care treatment utilization was higher in the majority of FIT hospitals compared to routine care during the first patient year. PIA contacts were higher in some and lower in some in the first and second patient years. Furthermore, the number of day-care days and PIA contacts seemed, as expected, to be inversely correlated, even though the correlation was not statistically significant. One reason for not reaching statistical significance could be the lower power as only 12 data points instead of 14 could be included with data for the CAPs missing. The effect size was, nonetheless, strong for the first patient year. Due to different treatment concepts in the FIT hospitals, reduced inpatient days were presumably compensated either by an increase in day-care treatment days or by more PIA contacts. Therefore, a significant overall effect on both target parameters could not be assumed. Three FIT hospitals (D, G and H), on the other hand, increased the utilization in both day-care and PIA treatment. This suggests a strengthening of both settings in these FIT hospitals. Qualitative research has also shown that the implementation of a GTB for a shift from in- to outpatient treatment (within the hospital) is crucial for the process of change [23].

The effects were strongest in the first patient year. Even though some effects might seem small in number, they imply desired changes for the health care system. For example, a reduction of 5.1 inpatient days in the first and 1.1 in days in the second year could be extrapolated to all psychiatric hospitals in Germany. This would result in about 13,000 fewer inpatient beds [50]. These resources could be used for patient-tailored treatment, such as PIA, day-care or home treatment.

#### Treatment continuity

Treatment continuity within the psychiatric sector increased more strongly in the majority of the FIT hospitals compared to routine care. Across all FIT hospitals, greater treatment continuity was evident. Since the PIA was also taken into account considering treatment continuity, a strengthening of the PIA can also lead to a strengthening of treatment continuity. In two FIT hospitals, however, the number of PIA contacts did not increase, but treatment continuity was strengthened. The individual reports of each FIT hospital [35] showed that the contact to a resident medical specialist for adult or child and adolescent psychiatry within the 30 days after hospital stay was higher in one of the two hospitals. The other hospital showed higher use of PIA and psychotherapist for a contact within 30 days. This indicates that there is an additional effect of enhancing treatment continuity among some FIT programs and that even if the number of contacts in PIA decreased, PIA treatment could still be used to offer a “bridging” treatment following discharge from inpatient treatment. As mentioned above, one of the goals of the law §64b SGB V is to overcome the strong fragmentation in the German psychiatric health care system. However, all FIT hospitals evaluated here focused on the treatment within the hospital itself. Our definition of treatment continuity, nonetheless, included both cross-sectoral (contact with resident physicians outside hospital setting) and cross-setting (contact with PIA) collaboration. Cross-sectoral treatment continuity was strengthened in some FIT hospitals, while in other FIT hospitals more cross-setting treatment continuity was visible. Cross-setting treatment continuity, nonetheless, did not need to include the same personnel in our analyses. Information on who treated the patient was not available in our dataset. Other studies, however, showed increased continuity of care with the same personnel among one FIT hospital compared to routine care [8, 51]. Enhancing continuity of treatment, in both cross-sectoral and cross-setting collaboration, is a key objective among all FIT hospitals, along with increasing the number of non-inpatient services. Schwarz et al. [23] also acknowledges that GBTs only partially address the problem of fragmentation in the German psychiatric health care system as long as they are limited to the hospital sector.

### Influence of CAP, FIT-like pre-existing contracts and first patient year

We decided to conduct sub-group meta-analyses excluding patients treated in CAP and only considering CAP patients, as the treatment, the conditions and circumstances of psychiatric treatment of children and youth differ from those of adults, and hence need separate consideration. Excluding all patients treated in CAP showed minor effects on the overall meta-analysis results. However, the pooled estimate for treatment continuity, which just reached statistical significance in the overall meta-analysis, was no longer significant excluding CAP. The main reason for the non-significance in the sub-group analysis is the broader confidence interval, which is due to the missing two units (two CAPs), hence a loss in statistical power. Due to the low number of FIT hospitals with CAP, we did not conduct separate analyses on CAP only. As the effects in CAPs, however, might different from adult psychiatry, further evaluations on FIT hospitals including CAP or on solely CAP are needed.

Previous analyses [28, 29, 35] on the effectiveness of FIT hospitals considering only patients being treated within the first year after FIT program onset showed a clear difference between those FIT hospitals that freshly started from routine care and those with a FIT-like pre-existing contract prior to FIT start. In contrast, our analyses including patients with study inclusion in the first three years showed no evidence of different effectiveness for FIT hospitals with and without a precursor contract. The analysis presented here was the first to be able to present results including patients from more than just the first year after FIT program start. Thus, new projects, i.e., without FIT-like precursor contracts, appear to be similar to those with precursor contracts within the first three FIT years with regard to the parameters examined here. Von Peter et al. [26] also stated that there seems to be an introductory phase when initiating new FIT programs. The degree of FIT implementation showed an effect, and staff evaluations were higher for departments with at least two years of history of FIT activity [26].

The largest effects were obvious in the first patient year with highest treatment needs during this initial treatment phase compared to later treatments. As the absolute numbers were higher, the relative difference could also be larger. Potential disincentives of FIT hospitals could have been to provide less inpatient together with less day-care and outpatient care as funding was already provided, and that patients might not receive adequate treatment after hospital discharge. However, this was not the case looking at treatment continuity. Patients were better integrated in the outpatient system compared to routine care. Nonetheless, hurdles for a stronger cooperation between hospitals and outpatient care providers are still present. Current FIT contracts only focus on treatment within FIT hospitals. Due to the historically strong separation of these sectors, a smoother cooperation between the sectors to assist patients navigating in the health care system is urgently needed.

### Strengths and limitations

The evaluation EVA64 is the largest scientific evaluation of its kind in the psychiatric field in Germany. Our analyses are based on data from more than 70 SHI funds and on more than 36,000 patients, each with an observation period of three years (one year before plus two years after study inclusion). Furthermore, we evaluated twelve FIT hospitals using the same method and compared their results. This is the first study that analyzes the results of patients being included in the first three years after FIT program start. This allowed investigating the change in those FIT hospitals that started from routine care. Further, we included almost all psychiatric diagnoses portraying the whole spectrum of psychiatric treatment vs. only focusing on one or two diagnostic groups. The EVA64 study also focusses on other outcomes of FIT hospitals compared to routine care, i.e. sick leave, discontinuation of contacts, physician and hospital hopping, re-admission rate, comorbidity, mortality, disease progression, guideline adherence, costs and cost-effectiveness [37]. Their results are provided on a FIT hospital single basis [35] (German only) and are not in the focus of this manuscript, but results across FIT hospitals will be published separately. Even though the consideration of children and youth were emphasized by law [18], only few FIT programs were implemented in CAPs. We could only include two CAPs in this analysis, and hence draw no firm conclusion for younger patients yet. Further research is needed here. A comprehensive evaluation of the FIT program of another CAP has already started [52].

Looking at the effectiveness of FIT hospitals, claims data offer strong evidence concerning the parameters analyzed here. SHI data deliver a complete and unbiased, prospective and patient-related image of in- and outpatient utilization (including PIA) [36]. However, information on patient-centered outcomes such as quality of life, treatment satisfaction or disease severity cannot be drawn from claims data. Further research is necessary here as only the combination of both perspectives can answer whether the changes in the mode of treatment found here are accompanied by more, for example, treatment satisfaction or less psychosocial impairment. In the end, both perspectives and the strengths of both are inevitable as supported by further studies [53]. In addition, the available data did not allow looking at increased collaboration between different settings within the hospital or at change of rehabilitation utilization. We investigated cross-sectoral treatment continuity, which was the focus of the FIT hospitals. Furthermore, the specific components of FIT hospitals that lead to changes in mode of patient treatment within hospitals such as continuity of treatment team or multi-professional cooperation and the factors that lead to FIT program adoption are not in the focus of this analysis and can be found elsewhere [7, 19].

## Conclusions

Our analysis suggests the effectiveness of FIT programs among adults considering the parameters analyzed here. However, due to the heterogeneity of treatment concepts, framework conditions and design of the individual contracts, the individual evaluation of each FIT hospital is still important. Nonetheless, this cross-FIT hospital analysis compares and summarizes the individual results and allows for a cross-FIT hospital overview. The results presented here generally recommend the continuation of the model projects (GBT & FIT), taking into account individual concepts and local conditions. Comparable changes in financing structures could also affect treatment routines in other countries.

### Supplementary Information

**Additional file 1: Figure S1.** Change in inpatient days, first and second patient year, FIT vs. routine hospitals, without CAP.

**Additional file 2: Figure S2.** Change in number of PIA contacts, first and second patient year, FIT vs. routine hospitals, without CAP.

**Additional file 3: Figure S3.** Change in treatment continuity, first and second patient year, FIT vs. routine hospitals, Odds Ratios, without CAP.

**Additional file 4: Table S1.** Description, inpatient days by group and year.

**Additional file 5: Table S2.** Description, day-care days by group and year.

**Additional file 6: Table S3.** Description, PIA contacts by group and year.

**Additional file 7: Table S4.** Description, Treatment continuity by group and year.

## Data Availability

The datasets generated and/or analyzed during the current study are not publicly available due to license agreements for the current study. For any question or request, please contact the corresponding author.

## Abbreviations

CAP: Department of child and adolescent psychiatry
DiD: Difference in Difference
FIT: Flexible and integrated treatment
GBT: Global treatment budget
PIA: Psychiatric outpatients department
SGB: Social Security Code (Sozialgesetzbuch)
SHI: Statutory health insurance
RC: Routine care

## References

[1] Thornicroft G, Tansella M (2004). Components of a modern mental health service: a pragmatic balance of community and hospital care: overview of systematic evidence. Br J Psychiatry.

[2] Salize HJ, Rossler W, Becker T (2007). Mental health care in Germany: current state and trends. Eur Arch Psychiatry Clin Neurosci.

[3] Busse R, Blumel M, Knieps F, Barnighausen T (2017). Statutory health insurance in Germany: a health system shaped by 135 years of solidarity, self-governance, and competition. Lancet.

[4] Nolting HD, Hackmann T. Bestandsaufnahme Von Komplexen Lokalen, regionalen und überregionalen sektorübergreifenden Modellprojekten Zur Versorgung Von Menschen Mit Psychischen Erkrankungen - Abschlussbericht [Inventory of complex local, regional and supra-regional cross-sectoral model projects for the care of people with mental illnesses - final report]. Berlin: IGES Institut GmbH; 2012.

[5] Wasem J, Reifferscheid A, Südmersen C, Faßbender R, Thomas D. Das pauschalierende Entgeltsystem für psychiatrische und psychosomatische Einrichtungen: Prüfung der Eignung alternativer Abrechnungseinheiten gemäß dem gesetzlichen Prüfauftrag nach § 17d Abs. 1 S. 2 KHG [The flat-rate remuneration system for psychiatric and psychosomatic facilities, review of the suitability of alternative billing units in accordance with the statutory review mandate pursuant to Section 17d (I) p. 2 KHG2012], IBES Discussion Paper, No. 195, Universität Duisburg-Essen, Institut für Betriebswirtschaft und Volkswirtschaft (IBES), Essen. 2012. https://www.econstor.eu/bitstream/10419/66142/1/729473201.pdf. Accessed 11 Jan 2024.

[6] Kliemt R, Häckl D, Klauber J, Geraedts M, Friedrich J, Wasem J, Beivers A (2020). Anreize Und Weiterentwicklungsperspektiven Der Vergütung Von Psychiatrie Und Psychosomatik Unter Der Berücksichtigung Von Modellprojekten [Incentives and further development perspectives of the remuneration of psychiatry and psychosomatics under consideration of model projects]. Krankenhaus-Report 2020 - finanzierung und Vergütung am Scheideweg.

[7] Afraz F, Vogel A, Dreher C, Berghöfer A (2021). Promoting Integrated Care through a Global Treatment Budget. Int J Integr care.

[8] Assheuer M, Beine K, Mehl C, Kellner M, Agelink M, Sieberer M (2021). [Implementation of continuity of care in Everyday Care - A comparison between two Psychiatric hospitals]. Psychiatr Prax.

[9] Sachverständigenrat zur Begutachtung der Entwicklung im Gesundheitswesen 2018. Koordinierte Versorgung von Menschen mit psychischen Erkrankungen [Coordinated care for people with mental illness]. Bonn/Berlin. 18. October 2018. ISBN: 978-3-95466-421-4. https://www.svr-gesundheit.de/fileadmin/Gutachten/Gutachten_2018/Gutachten_2018.pdf. Accessed 11 Jan 2024.

[10] Blumel M, Spranger A, Achstetter K, Maresso A, Busse R (2020). Germany: Health System Review. Health Syst Transit.

[11] Deister A, Zeichner D, Roick C (2004). Ein Regionales Budget für die psychiatrie. Erste Erfahrungen Aus Einem Modellprojekt [A Regional Budget for Psychiatry. First experiences from a model project]. Psychoneuro.

[12] Stierlin AS, Herder K, Helmbrecht MJ, Prinz S, Walendzik J, Holzmann M (2014). Effectiveness and efficiency of integrated mental health care programmes in Germany: study protocol of an observational controlled trial. BMC Psychiatry.

[13] Lambert M, Bock T, Daubmann A, Meigel-Schleiff C, Lange B, Lange M (2014). [The Hamburg-model of integrated care for patients with psychosis: part 1. Rationale, treatment concept and results of the pre-study]. Psychiatr Prax.

[14] Konig HH, Heinrich S, Heider D, Deister A, Zeichner D, Birker T (2010). [The regional psychiatry budget (RPB): a model for a new payment system of hospital based mental health care services]. Psychiatr Prax.

[15] Roick C, Deister A, Zeichner D, Birker T, Konig HH, Angermeyer MC (2005). [The regional budget for mental health care: a new approach to combine inpatient and outpatient care]. Psychiatr Prax.

[16] Roick C, Heinrich S, Deister A, Zeichner D, Birker T, Heider D (2008). Das Regionale Psychiatriebudget: Kosten Und Effekte eines neuen sektorübergreifenden Finanzierungsmodells für die psychiatrische versorgung [The regional psychiatry budget: costs and effects of a new multisector financing model for psychiatric care]. Psychiatr Prax.

[17] Deister A, Zeichner D, Witt T, Forster HJ (2010). [Changes in mental health care by a regional budget: results of a pilot project in Schleswig-Holstein (Germany)]. Psychiatr Prax.

[18] Federal Ministry of Justic and Consumer Protection. § 64b SGB V: Modellvorhaben zur Versorgung psychisch kranker Menschen [Modell projects for the care of mentally ill people] 2012 . Available from: https://www.gesetze-im-internet.de/sgb_5/__64b.html. Cited 2023.

[19] Johne J, von Peter S, Schwarz J, Timm J, Heinze M, Ignatyev Y. Evaluation of new flexible and integrative psychiatric treatment models in Germany-assessment and preliminary validation of specific program components. BMC Psychiatry. 2018;18:278–87. 10.1186/s12888-018-1861-1 PMC6122621 30176836

[20] Jacobs R, Chalkley M, Aragon MJ, Bohnke JR, Clark M, Moran V (2018). Funding approaches for mental health services: is there still a role for clustering? BJPsych. Adv.

[21] Schroder B, Flessa S (2017). [Regional budgets in Psychiatry: an alternative to hospital per Diem charges and the new reimbursement system? - a case study from the District of Dithmarschen]. Psychiatr Prax.

[22] GKV Spitzenverband, Verband der Privaten Krankenversicherung e.V., Deutsche Krankenhausgesellschaft e.V. Gemeinsamer Bericht zur Einführung eines pauschalisierenden Entgeltsystems für psychiatrische und psychosomatische Einrichtungen [Joint report on the introduction of a flat-rate remuneration system for psychiatric and psychosomatic facilities]. 2019. https://dserver.bundestag.de/btd/19/128/1912850.pdf. Accessed 11 Jan 2024.

[23] Schwarz J, Galbusera L, Bechdolf A, Birker T, Deister A, Duve A (2020). Changes in German Mental Health Care by implementing a global treatment Budget-A mixed-method process evaluation study. Front Psychiatry.

[24] Schwarz J, Zeipert M, Ignatyev Y, Indefrey S, Rehr B, Timm J (2020). Implementation and stakeholders’ experiences with Home Treatment in Germany’s Integrative and Flexible Psychiatric Care models - a mixed-methods study. Psychother Psychosom Med Psychol.

[25] Wheeler C, Lloyd-Evans B, Churchard A, Fitzgerald C, Fullarton K, Mosse L (2015). Implementation of the Crisis Resolution Team model in adult mental health settings: a systematic review. BMC Psychiatry.

[26] von Peter S, Ignatyev Y, Johne J, Indefrey S, Kankaya OA, Rehr B, et al. Evaluation of flexible and integrative psychiatric treatment models in Germany—A mixed-method patient and staff-oriented exploratory study. Front Psychiatry. 2019;9(785). 10.3389/fpsyt.2018.00785 PMC6349706 30723433

[27] Mueller-Stierlin AS, Helmbrecht MJ, Herder K, Prinz S, Rosenfeld N, Walendzik J (2017). Does one size really fit all? The effectiveness of a non-diagnosis-specific integrated mental health care program in Germany in a prospective, parallel-group controlled multi-centre trial. BMC Psychiatry.

[28] Baum F, Schoffer O, Neumann A, Seifert M, Kliemt R, March S (2020). Effectiveness of Global Treatment Budgets for patients with Mental disorders—Claims Data Based Meta-Analysis of 13 controlled studies from Germany. Front Psychiatry.

[29] Neumann A, Baum F, Seifert M, Schoffer O, Kliemt R, March S (2021). [Reduction of days in Inpatient Care in Psychiatric hospitals with flexible and Integrated Treatment for patient-centered care with a Global Budget - results with three-year follow-up from the evaluation study EVA64]. Psychiatr Prax.

[30] Baum F, Schmitt J, Seifert M, Kliemt R, Kubat D, March S (2022). Lengths of inpatient stay and sick leave of patients with mental diseases: disorder-specific effects of flexible and integrated treatment programs in Germany. Transl Psychiatry.

[31] Konig HH, Heider D, Rechlin T, Hoffmann P, Birker T, Heinrich S (2013). [How does the Regional Psychiatry Budget (RPB) work in an area with initially low capacity of psychiatric hospital beds?]. Psychiatr Prax.

[32] Indefrey S, Braun B, von Peter S, Bechdolf A, Birker T, Duve A (2020). Implementation of a Global Treatment Budget in Psychiatric Departments in Germany-results and critical factors for Success from the Staff Perspective. Front Psychiatry.

[33] Schmid P, Steinert T, Borbe R (2013). [Implementing models of cross-sectoral mental health care (integrated health care, regional psychiatry budget) in Germany: systematic literature review]. Psychiatr Prax.

[34] Federal Ministry of Justice and Consumer Protection. § 65 SGB V: Auswertung der Modellvorhaben [Evaluation of model projects]. 2012. Available from: https://www.sozialgesetzbuch-sgb.de/sgbv/65.html. Cited 2023.

[35] Study Group EVA64. Evaluation of FIT hospitals (Evaluation von Modellvorhaben in der Psychiatrie nach § 64b SGB V) - Reports. 2022. Available from: https://www.uniklinikum-dresden.de/de/das-klinikum/universitaetscentren/zegv/projekte/eva64. Cited 2021 31 May 2022.

[36] Swart E, Amelung V, Stein V, Goodwin V, Balicer R, Nolte E, Suter E (2021). Claims data for evaluation. Handbook Integrated Care.

[37] Neumann A, Swart E, Häckl D, Kliemt R, March S, Kuster D et al. The influence of cross-sectoral treatment models on patients with mental disorders in Germany: study protocol of a nationwide long-term evaluation study (EVA64). BMC Psychiatry. 2018;18. 10.1186/s12888-018-1721-z PMC5960179 29776348

[38] Petzold T, Neumann A, Seifert M, Kuster D, Pfennig A, Weiss J (2019). [Identification of control hospitals for the implementation of the Nationwide and standardized evaluation of Model projects according to section sign 64b SGB V: analysis of data from structured Quality Reports]. Gesundheitswesen.

[39] Hoffmann W, Latza U, Baumeister SE, Brunger M, Buttmann-Schweiger N, Hardt J (2019). Guidelines and recommendations for ensuring good epidemiological practice (GEP): a guideline developed by the German Society for Epidemiology. Eur J Epidemiol.

[40] Swart E, Gothe H, Geyer S, Jaunzeme J, Maier B, Grobe T (2015). Gute Praxis Sekundärdatenanalyse (GPS): Leitlinien Und Empfehlungen [Goode Practice Secondary Data Analysis: guidelines and recommendations]. Gesundheitswesen.

[41] Swart E, Bitzer EM, Gothe H, Harling M, Hoffmann F, Horenkamp-Sonntag D (2016). A Consensus German Reporting Standard for Secondary Data Analyses, Version 2 (STROSA-STandardisierte BerichtsROutine fur SekundardatenAnalysen). Gesundheitswesen.

[42] Quan H, Sundararajan V, Halfon P, Fong A, Burnand B, Luthi JC (2005). Coding algorithms for defining comorbidities in ICD-9-CM and ICD-10 administrative data. MedCare.

[43] Falkai PH (2013). S3-Leitlinie Psychosoziale Therapien bei schweren psychischen Erkrankungen [S3-Guideline Psycho-Social Therapies for Severe Mental Illnesses].

[44] Viechtbauer W (2010). Conducting Meta-analyses in R with the metafor Package. J Stat Softw.

[45] Trikalinos TA, Olkin I (2012). Meta-analysis of effect sizes reported at multiple time points: a multivariate approach. Clin Trials.

[46] R Core Team (2012) R: A Language and Environment for Statistical Computing. Vienna: R Foundation for Statistical Computing. http://www.r-project.org/. Accessed 11 Jan 2024.

[47] Ziguras SJ, Stuart GW (2000). A meta-analysis of the effectiveness of mental health case management over 20 years. Psychiatr Serv.

[48] Lambert M, Bock T, Schottle D, Golks D, Meister K, Rietschel L (2010). Assertive community treatment as part of integrated care versus standard care: a 12-month trial in patients with first- and multiple-episode schizophrenia spectrum disorders treated with quetiapine immediate release (ACCESS trial). J Clin Psychiatry.

[49] Ignatyev Y, Mundt AP, von Peter S, Heinze M (2019). Hospital length of stay among older people treated with flexible and integrative psychiatric service models in Germany. Int J Geriatr Psychiatry.

[50] Schmedders M, Neubert O (2023). Transfer of model projects according to § 64b SGB V to standard care. Nervenheilkunde.

[51] de Cruppé W, Assheuer M, Geraedts M, Beine K (2023). Association between continuity of care and treatment outcomes in psychiatric patients in Germany: a prospective cohort study. BMC Psychiatry.

[52] Neumann A, Hense H, Baum F, Kliemt R, Seifert M, Harst L (2021). Evaluation of a flexible and integrative psychiatric care model in a department of child and adolescent psychiatry in Tubingen, Germany: study protocol (EVA_TIBAS). BMC Health Serv Res.

[53] Soltmann B, Neumann A, March S, Weinhold I, Hackl D, Kliemt R (2021). Multiperspective and Multimethod evaluation of Flexible and Integrative Psychiatric Care Models in Germany: study protocol of a prospective, controlled Multicenter Observational Study (PsychCare). Front Psychiatry.

