# Supplementary material for: Changes in patient care through flexible and integrated treatment programs in German psychiatric hospitals: meta-analyses based on a series of controlled claims-based cohort studies
Source: BMC Psychiatry. 2024 Jan 26;24:74. doi: 10.1186/s12888-024-05500-0 (PMC10811876; doi:10.1186/s12888-024-05500-0)
Supplement: Supplementary file 3 — Additional file 3: Figure S3. Change in treatment continuity, first and second patient year, FIT vs. routine hospitals, Odds Ratios, without CAP. [file 12888_2024_5500_MOESM3_ESM.docx]

**supplementary file to**

Changes in patient care through flexible and integrated treatment programs in German psychiatric hospitals: meta-analyses based on a series of controlled claims-based cohort studies

Anne Neumann^*1^, Jochen Schmitt^1^, Martin Seifert^1^, Roman Kliemt^2^, Stefanie March^3, 4^, Dennis Häckl^2^, Enno Swart^3^, Andrea Pfennig^5^, Fabian Baum^1^

^1^Center of Evidence-based Health Care, Medizinische Fakultät Carl Gustav Carus, Technische Universität Dresden, Germany

^2^WIG2 Scientific Institute for Health Economics and Health System Research Leipzig, Germany

^3^Institute of Social Medicine and Health Services Research, Medical Faculty, Otto-von-Guericke- University Magdeburg, Germany

^4^Hochschule Magdeburg-Stendal, Department of Social Work, Health and Media, Germany

^5^Department of Psychiatry and Psychotherapy, Carl Gustav Carus University Hospital, Technische Universität Dresden, Germany


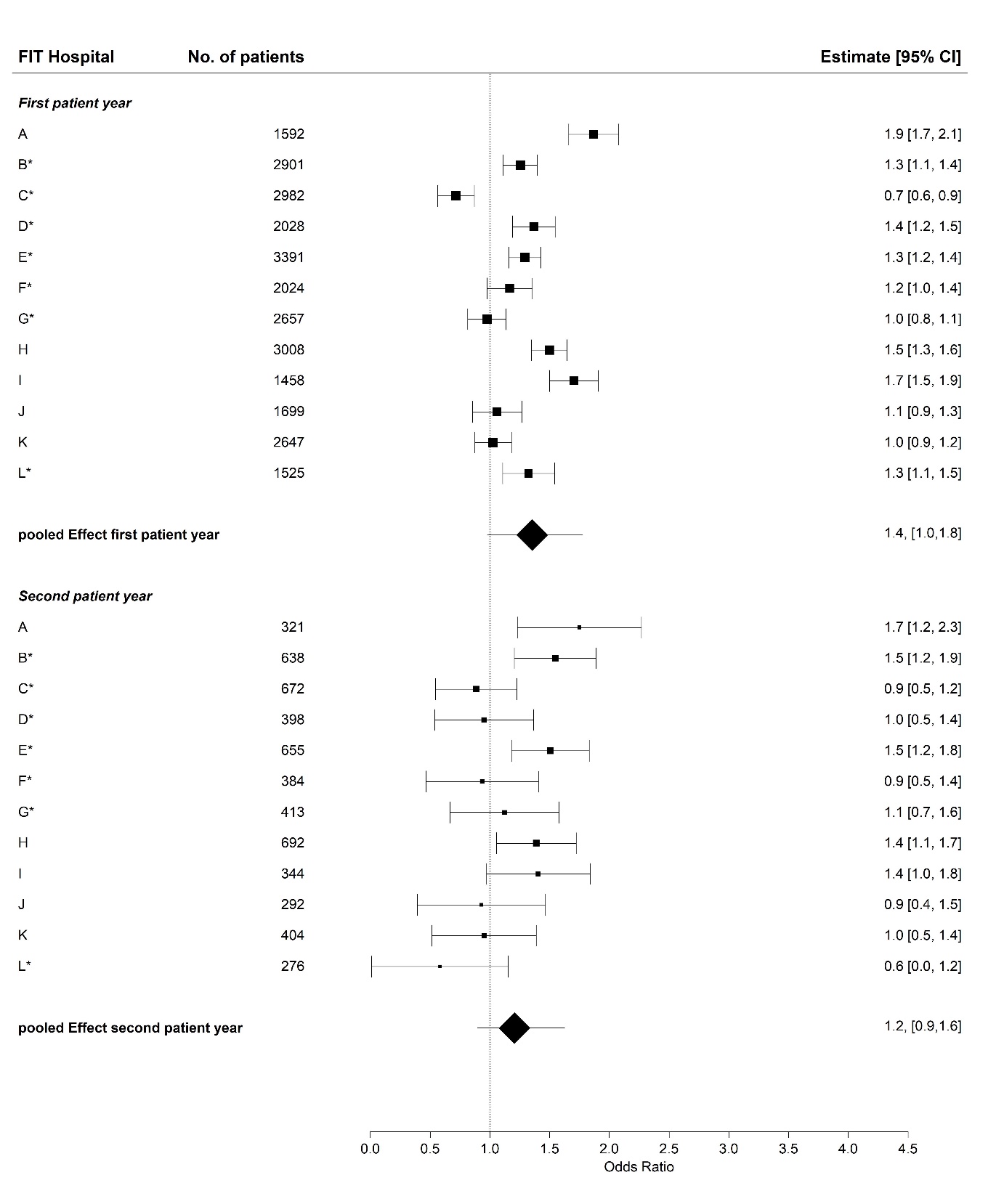

**Figure S3: Change in treatment continuity, first and second patient year, FIT vs. routine hospitals, Odds Ratios, without CAP**

*Treatment continuity = outpatient treatment within 30 days after hospital discharge
* = FIT hospital with FIT-like pre-existing contract
CAP = Department of child and adolescent psychiatry
Difference in Difference = The difference in difference (DiD) estimate compares the average change in the outcome over time for the FIT hospital in comparison to the average change in time in routine care. Thus, greater increase over time in the FIT hospital compared to routine care are associated with a positive DiD estimate and vice versa.*
