# Supplementary material for: Changes in patient care through flexible and integrated treatment programs in German psychiatric hospitals: meta-analyses based on a series of controlled claims-based cohort studies
Source: BMC Psychiatry. 2024 Jan 26;24:74. doi: 10.1186/s12888-024-05500-0 (PMC10811876; doi:10.1186/s12888-024-05500-0)
Supplement: Supplementary file 6 — Additional file 6: Table S3. Description, PIA contacts by group and year. [file 12888_2024_5500_MOESM6_ESM.docx]

**supplementary file to**

Changes in patient care through flexible and integrated treatment programs in German psychiatric hospitals: meta-analyses based on a series of controlled claims-based cohort studies

Anne Neumann^*1^, Jochen Schmitt^1^, Martin Seifert^1^, Roman Kliemt^2^, Stefanie March^3, 4^, Dennis Häckl^2^, Enno Swart^3^, Andrea Pfennig^5^, Fabian Baum^1^

^1^Center of Evidence-based Health Care, Medizinische Fakultät Carl Gustav Carus, Technische Universität Dresden, Germany

^2^WIG2 Scientific Institute for Health Economics and Health System Research Leipzig, Germany

^3^Institute of Social Medicine and Health Services Research, Medical Faculty, Otto-von-Guericke- University Magdeburg, Germany

^4^Hochschule Magdeburg-Stendal, Department of Social Work, Health and Media, Germany

^5^Department of Psychiatry and Psychotherapy, Carl Gustav Carus University Hospital, Technische Universität Dresden, Germany

Table S3: Description, PIA contacts by group and year

| **FIT hospital** | **PIA contacts (average of all patients included)** | | | | | |
| --- | --- | --- | --- | --- | --- | --- |
|  | FIT | | | RC | | |
|  | -1 year | 1^st^ year | 2^nd^ year | -1 year | 1^st^ year | 2^nd^ year |
| **A** | 0.1 | 6.2 | 4.0 | 0.2 | 2.6 | 1.7 |
| **B** | 0.2 | 8.5 | 3.0 | 0.3 | 2.9 | 1.5 |
| **C** | 0.0 | 2.5 | 2.0 | 0.2 | 4.2 | 2.6 |
| **D** | 0.0 | 3.6 | 1.6 | 0.1 | 2,7 | 1.5 |
| **E** | 0.1 | 2.2 | 1.0 | 0.0 | 3.5 | 2.2 |
| **F** | 0.1 | 2.1 | 1.0 | 0.1 | 3.7 | 2.2 |
| **G** | 0.1 | 3.8 | 2.3 | 0.1 | 3.2 | 2.0 |
| **H** | 0.1 | 3.9 | 1.7 | 0.2 | 2.7 | 1.5 |
| **I** | 0.1 | 5.3 | 2.7 | 0.1 | 3.4 | 1.8 |
| **J** | 0.3 | 2.5 | 1.6 | 0.1 | 2.6 | 1.8 |
| **K** | 0.0 | 2.3 | 1.2 | 0.2 | 3.2 | 1.9 |
| **L** | 0.0 | 1.9 | 1.9 | 0.3 | 3.3 | 2.2 |
| **J - CAP** | 0.0 | 3.3 | 1.3 | 0.0 | 6.4 | 2.1 |
| **L - CAP** | 0.0 | 3.9 | 1.6 | 0.1 | 3.4 | 1.4 |

*PIA = psychiatric outpatient department
FIT = flexible and integrated treatment = those hospitals with innovative financing and treatment forms (intervention group); RC = routine care
-1 year = one patient year before study inclusion; 1^st^ yr. = first patient year; 2^nd^ yr. = second patient year
CAP = Department of child and adolescent psychiatry*
