# Supplementary material for: Changes in patient care through flexible and integrated treatment programs in German psychiatric hospitals: meta-analyses based on a series of controlled claims-based cohort studies
Source: BMC Psychiatry. 2024 Jan 26;24:74. doi: 10.1186/s12888-024-05500-0 (PMC10811876; doi:10.1186/s12888-024-05500-0)
Supplement: Supplementary file 7 — Additional file 7: Table S4. Description, Treatment continuity by group and year. [file 12888_2024_5500_MOESM7_ESM.docx]

**supplementary file to**

Changes in patient care through flexible and integrated treatment programs in German psychiatric hospitals: meta-analyses based on a series of controlled claims-based cohort studies

Anne Neumann^*1^, Jochen Schmitt^1^, Martin Seifert^1^, Roman Kliemt^2^, Stefanie March^3, 4^, Dennis Häckl^2^, Enno Swart^3^, Andrea Pfennig^5^, Fabian Baum^1^

^1^Center of Evidence-based Health Care, Medizinische Fakultät Carl Gustav Carus, Technische Universität Dresden, Germany

^2^WIG2 Scientific Institute for Health Economics and Health System Research Leipzig, Germany

^3^Institute of Social Medicine and Health Services Research, Medical Faculty, Otto-von-Guericke- University Magdeburg, Germany

^4^Hochschule Magdeburg-Stendal, Department of Social Work, Health and Media, Germany

^5^Department of Psychiatry and Psychotherapy, Carl Gustav Carus University Hospital, Technische Universität Dresden, Germany

Table S4: Description, Treatment continuity by group and year

| **FIT hospital** | **Treatment continuity (%)** | | | |
| --- | --- | --- | --- | --- |
|  | FIT | | RC | |
|  | 1^st^ year | 2^nd^ year | 1^st^ year | 2^nd^ year |
| **A** | 39.6 | 39.7 | 27.7 | 32.0 |
| **B** | 35.0 | 36.1 | 24.3 | 26.4 |
| **C** | 13.6 | 14.7 | 17.1 | 17.3 |
| **D** | 22.0 | 20.4 | 16.6 | 21.8 |
| **E** | 19.6 | 21.3 | 17.0 | 18.8 |
| **F** | 17.1 | 17.4 | 15.2 | 13.3 |
| **G** | 16.0 | 18.6 | 15.8 | 18.1 |
| **H** | 31.2 | 33.5 | 20.6 | 24.7 |
| **I** | 21.3 | 29.2 | 17.2 | 20.7 |
| **J** | 22.6 | 24.8 | 23.6 | 25.8 |
| **K** | 24.7 | 26.0 | 24.7 | 34.2 |
| **L** | 22.9 | 17.7 | 19.6 | 26.3 |
| **J - CAP** | 19.4 | 21.2 | 24.4 | 27.4 |
| **L - CAP** | 13.4 | 37.0 | 19.1 | 17.5 |

*Treatment continuity = outpatient treatment within 30 days after hospital discharge
FIT = flexible and integrated treatment = those hospitals with innovative financing and treatment forms (intervention group); RC = routine care
1^st^ yr. = first patient year; 2^nd^ yr. = second patient year
CAP = Department of child and adolescent psychiatry*
